# Longitudinal Changes in Plasma GFAP and Their Potential Role in Alzheimer's Disease Progression

**DOI:** 10.1002/alz70856_104085

**Published:** 2025-12-24

**Authors:** Yung‐Shuan Lin, Yu‐Yang Hung, Wei‐Ju Lee, Jong‐Ling Fuh

**Affiliations:** ^1^ Taipei Veterans General Hospital, Taipei, Taiwan; ^2^ Taichung Veterans General Hospital, Taichung, Taiwan

## Abstract

**Background:**

Brain amyloid deposition may affect the relationship between plasma glial fibrillary acidic protein (GFAP), a marker of astrocytic activation, and cognitive decline, but its role in modulating cognitive outcomes remains unclear.

**Method:**

Participants across the Alzheimer's disease (AD) continuum (cognitively unimpaired [CU], mild cognitive impairment [MCI], and dementia) were recruited from two centers in Taiwan. Diagnoses were confirmed via consensus. Plasma GFAP levels were measured using Quanterix SIMOA kits at baseline, and MMSE scores were assessed at baseline and 1 year. Florbetaben 18F (FBB) amyloid PET scans were conducted in a subset (centiloid ≥30 indicating amyloid positivity).

**Result:**

The study included 236 participants (dementia: n = 95; MCI: n = 79; CU: n = 63; mean age: 70.8 ± 7.1; female: 51.7%; APOE ɛ4 carriers: 35.3%; baseline GFAP: 271.8 ± 189.1 pg/uL). GFAP levels were significantly associated with cognitive status, age, and APOE ɛ4 status. Adjusted analyses showed dementia patients had higher GFAP levels than CU (*B* = 123.75, *p* < 0.001). Follow‐up assessments (*n* = 175) after 1.2 ± 0.7 years showed no direct association between GFAP levels and MMSE change. Among 83 participants with FBB PET scans, amyloid‐positive (A+) cognitively impaired individuals (MCI and dementia) had higher GFAP levels than amyloid‐negative (A‐) CU (*p* < 0.001) and A‐ CI (*p* = 0.033). A significant interaction between GFAP and amyloid status was identified (*B* = ‐0.005, *p* =  0.0192).

**Conclusion:**

Plasma GFAP levels are associated with age, APOE ɛ4 status, and cognitive status, with higher levels in dementia and amyloid‐positive individuals. GFAP may better predict cognitive decline in A+ individuals, suggesting a synergy between neuroinflammation and amyloid pathology. Further research is needed to investigate this interaction.

